# Longitudinal modeling of Post-COVID-19 condition over three years: A machine learning approach using clinical, neuropsychological, and fluid markers

**DOI:** 10.1038/s41598-026-37635-3

**Published:** 2026-02-14

**Authors:** Julia Walders, Sophie Wetz, Ana Sofia Costa, Anna Hofmann, Jörg B. Schulz, Kathrin Reetz, Ravi Dadsena

**Affiliations:** 1 Department of Neurology, RWTH Aachen University, Pauwelsstraße 30, 52074 Aachen, Germany; 2 JARA Brain Institute Molecular Neuroscience and Neuroimaging (INM-11), Research Centre Jülich and RWTH Aachen University, 52056 Aachen, Germany; 3 German Center for Neurodegenerative Diseases (DZNE), 72076 Tübingen, Germany; 4 Department of Cellular Neurology, Hertie Institute for Clinical Brain Research, University Hospital Tübingen, 72076 Tübingen, Germany

**Keywords:** Longitudinal data, Machine learning, Clinical biomarkers, Predictive modeling, Long COVID-19, Biomarkers, Computational biology and bioinformatics, Diseases, Medical research, Neurology, Neuroscience

## Abstract

Post-COVID-19 condition (PCC) manifests with prolonged, heterogeneous symptoms challenging both, diagnosis and therapeutic management. This three-year longitudinal study analyzed data from 93 adults (mean age of 48.9 ± 14.0, 60 female) after confirmed SARS-CoV-2 infection. Every follow-up visit included clinical, neuropsychological, and laboratory assessments, capturing multidimensional indicators of patient health. A machine learning framework was implemented to classify temporal stage of patient health status, identify visit-specific predictive markers, and manage incomplete data using both native handling in tree-based models and explicit imputation techniques. Gradient boosting methods consistently achieved the best performance across all visit comparisons, achieving F1-scores close to or above 90%. Classification performance improved with greater time intervals between visits, suggesting progressive divergence in patient phenotypes over time. For discriminating follow-up stages, inflammatory markers emerged as the most informative predictors, followed by SARS-CoV-2 antibody levels and neuropsychiatric measures for fatigue and cognitive performance. Interpretability analyses using SHAP and LIME confirmed the contribution of these features, while revealing shifts in feature relevance across years. These findings highlight the utility of machine learning in characterizing follow-up stage separability in PCC and offer clinically interpretable insights that prioritize immune and neuropsychological measures for monitoring and risk-stratified follow-up.

**Supplementary Information:**

The online version contains supplementary material available at 10.1038/s41598-026-37635-3.

## Introduction

While most people recover after acute COVID-19, 5–10% of individuals experience persistent and debilitating symptoms extending months or even years beyond the initial illness^1^. Without an alternative explanation this condition is referred to as Post-COVID-19 Condition (PCC) as defined by the World Health Organization (WHO)^2,3^. Understanding the long-term consequences of COVID-19 has become one of the most pressing challenges in public health research. Roughly 400 million people worldwide are estimated to have experienced long COVID, with the condition carrying an annual economic burden of around 1% of the global economy^4^. The symptoms are multifaceted and encompass chronic fatigue, a variety of cardiovascular and pneumological symptoms and neuropsychological impairment, presenting a great challenge for diagnosis, prognosis, and therapeutic intervention. In the absence of biomarkers and relying only on temporal criteria, the WHO definition remains vague, making it difficult to identify cases in real-world data, critical for effective patient management and public health planning. Data capturing the dynamic interplay of factors influencing disease progression over extended time periods is limited up to date. The complex and non-linear relationships between various clinical presentations, laboratory abnormalities, and cognitive performance necessitate advanced analytical approaches capable of discerning subtle patterns and predicting future outcomes^1,2^.

Machine learning techniques, with their capacity to model non-linear relationships and adapt to heterogeneous data sources, are increasingly used to explore prognostic patterns in COVID-19 survivors and refine clinical phenotyping. Recent advances in artificial intelligence and machine learning have shown considerable promise for detection, grading and prediction of PCC by modelling high-dimensional clinical and imaging-based data^5,6^. Azhir et al. for example developed an attention-based algorithm that accounts for chronic conditions and differential diagnoses, outperforming ICD-10 code U09.9, reducing bias, and enabling better research and patient care^7^. Other studies have demonstrated the utility of machine learning including deep neural networks to predict disease outcome of COVID-19 severity and to identify risk factors of PCC, offering novel perspectives on disease mechanisms and possible therapeutic targets^8–11^. One study has leveraged deep learning and data-driven modeling to predict functional impairments and work capacity reductions in PCC patients, further highlighting the potential of artificial intelligence to capture subtle long-term clinical dynamics^12^. Recent systematic reviews have further confirmed the growing evidence base for artificial intelligence applications in clinical practice^13–15^.

While these emerging studies underscore the growing role of artificial intelligence in PCC research, many prior investigations have focused on relatively short follow-up periods and single-domain data types, limiting their applicability for understanding chronic disease evolution. Moreover, the interpretability of machine learning models remains an ongoing challenge in clinical research, where transparent and explainable predictions are essential for clinical translation. To address these limitations, we present a comprehensive machine learning framework applied to a unique longitudinal cohort with three years of follow-up, integrating clinical, neuropsychological, and fluid biomarkers. Our approach combines multiple machine learning classifiers with validation schemes and modeling strategies to provide transparent and reproducible insights into how patient profiles differ across follow-up stages over time.

By using a complex longitudinal clinical dataset focusing on both, predictive performance and a multi-domain, multi-year perspective, this study aims to identify a set of markers that may support clinicians and researchers in making informed diagnostic decisions and in identifying and monitoring PCC patients who are suitable for distinct therapeutic options currently under investigation, such as immune-targeted treatments.

## Materials and methods

### Study participants

This monocentric, longitudinal observational study was conducted at the Department of Neurology, University Hospital RWTH Aachen (UKA), Germany. A total of 93 adult patients with confirmed SARS-CoV-2 infection were enrolled between August 2020 and March 2021 and followed annually across three consecutive years with four follow-up assessments. Eligibility criteria included age ≥ 18 years and persistent neurological or neuropsychological symptoms following acute COVID-19, consistent with the definition of PCC. All participants underwent standardized clinical, neuropsychological, and laboratory evaluations at each follow-up visit.

### Standard protocol approvals, registrations and patient consent

All procedures involving human participants were approved by the local ethics committee (“Ethikkommission an der Medizinischen Fakultät der RWTH Aachen”, reference number EK 192/20) and were conducted in accordance with the principles outlined in the Declaration of Helsinki. Written informed consent was obtained from all participants prior to inclusion in the study. The study was designed and reported in accordance with the STROBE (Strengthening the Reporting of Observational Studies in Epidemiology) guidelines to ensure methodological transparency and completeness in observational research.

### Procedures

#### Clinical and neuropsychological assessments

As previously described, we used standardized patient reported outcome measures at all study visits including the Fatigue Scale for Motor and Cognitive Functions (FSMC), the Hospital Anxiety and Depression Scale (HADS-D), the Epworth Sleepiness Scale (ESS), and the Pittsburgh Sleep Quality Index (PSQI)^16–19^. Additionally, in a structured interview, the presence of various, specific PCC symptoms was also investigated (Supplemental Table 1).

The Montreal Cognitive Assessment (MoCA) served as a brief screening tool. As previously published, the neuropsychological assessment included standardized measures for attention, information processing, and psychomotor speed (Trail Making Test-A, TMT-A; Alertness subtests of the Test of Attentional Performance, TAP; Symbol Digit Modalities Test, SDMT), executive functions (Stroop Test, phonemic and semantic verbal fluency; Digit Span backward; Trail Making Test-B, TMT-B), language (Naming tasks and phonemic and semantic verbal fluency), as well as memory and learning (Digit Span forward; Verbal Learning and Memory Test, VLMT)^16–19^.

### Fluid biomarker analysis

At each study visit blood samples were drawn and immediately analyzed by our in-house laboratory at the UKA and included hematological markers, inflammatory markers including cytokines, coagulation parameters, SARS-CoV-2 antibodies, metabolic markers, organ-specific functional parameters and vitamins. We also assessed neuronal injury markers including neurofilament light (NfL) and glial fibrillary acidic protein (GFAP). To this end a duplicate blood set was divided into 500-microliter aliquots and stored at − 80 °C in the centralized biomaterial bank at the faculty of medicine at RWTH Aachen university and later used for measurement of NFL and GFAP utilizing commercially available assay kits on the SIMOA HD-X patform at the hertie Institute for clinical brain research in Tübingen, Germany as previously described^18^. A complete list of all fluid markers assessed within our study can be found in supplemental table 2.

### Machine learning modeling

In this study, we implemented a comprehensive machine learning pipeline designed to classify the follow-up timepoint (temporal stage) from multimodal patient profiles of COVID-19 patients across follow-up visits. Our objective was to evaluate supervised classifiers that predict the follow-up timepoint a patient belongs to from clinical, neuropsychological, and laboratory features, and to identify the key variables driving this discrimination. The raw dataset included multiple modalities of information including clinical assessments, fluid markers, and neuropsychological measurements collected over three consecutive years and comprising four follow-up visits. For neuropsychological measures, we used z-scores to keep a consistent scale across visits while avoiding external demographic adjustments. Preprocessing of data began by first selecting all PCC patient records (no healthy controls); no additional filtering was performed prior to modeling. We ensured data cleanliness by standardizing numeric formats, addressing inconsistencies in decimal separators, and coercing any non-numeric entries to missing values, ensuring a harmonized numeric dataset suitable for model training.

The time component was discretized into four follow-up points after acute COVID-19, labeled as visit 1 (= 6 months), visit 2 (= 14 months), visit 3 (= 23 months), and visit 4 (= 38 months), according to the follow-up time index in the original dataset. For every pairwise combination of visits, for example comparing visit1 versus visit4 or visit2 versus visit3, we prepared binary classification tasks where the target label was assigned as one if the observation belonged to the latter year and zero otherwise. Prior to modeling, features were screened to exclude those with excessive missingness greater than 50%, as well as those with zero variance within the training subset, thereby preventing the inclusion of non-informative or potentially noisy predictors. To distinguish whether model performance reflected genuine clinical patterns versus merely temporal progression, we implemented a time-only baseline model for each pairwise comparison. This baseline model used solely the discrete time variable (visit index: 0, 1, 2, 3) as predictor, while clinical models explicitly excluded the time variable from the feature set. Both model types employed identical cross-validation strategies to ensure fair comparison.

Patients were grouped using their unique subject identifiers to avoid data leakage between training and validation phases, preserving the longitudinal integrity of the dataset. For model training and validation, we employed a subject wise Group K-Fold cross-validation strategy with five folds^20^. Given the limited sample size, no independent hold-out test set was used; instead, performance estimates were obtained directly from the cross-validation folds, ensuring that all observations from a single patient appeared exclusively in either the training or the validation set within any given fold. All preprocessing steps (feature screening, scaling), imputation, model fitting, and interpretation were performed within each training fold and applied to the corresponding validation fold only, to prevent information leakage. This approach provides a more realistic estimate of model performance on unseen patient data^20,21^.

Our modelling approach involved two primary stages. First, we applied direct modeling without prior imputation using tree-based ensemble methods known for their ability to internally handle missing values^22–24^. This included CatBoost, LightGBM, XGBoost, and Histogram Gradient Boosting classifiers^25,26^. These models are particularly advantageous in clinical data settings because they allow the inclusion of cases with partially missing data, thereby retaining a larger portion of the dataset and maintaining clinical relevance^27,28^. In the second stage, we applied data imputation strategies to manage missing values before training classifiers that do not natively handle missing entries. Two imputation techniques were implemented: k-nearest neighbors (KNN) imputation for continuous variables after standardization to zero mean and unit variance, and random forest (RF) imputation as an additional method to account for potential non-linear relationships in the missingness patterns^29,30^. Binary variables were imputed using the most-frequent value strategy^31^. To preserve information about the missing data mechanism itself, we generated missingness indicators for each feature, encoding whether a value was originally missing as an additional binary predictor^32^. Following imputation and feature scaling, zero-variance features were excluded within each fold to prevent the inclusion of non-informative predictors and reduce overfitting risk.

A diverse set of classifiers was then employed on the imputed datasets, broadening our evaluation to include not only tree-based methods but also classical machine learning algorithms such as Support Vector Machines, RF, Decision Trees, Multi-Layer Perceptron, and Naïve Bayes classifiers. Each classifier was trained on the preprocessed training data within each cross-validation fold and evaluated on the corresponding testing fold to ensure unbiased performance estimates.

### Model performance evaluation and feature interpretability

We assessed model performance across all classifiers and year wise comparisons using standard evaluation metrics^33^. Accuracy was calculated to measure the proportion of correctly predicted samples. Precision was used to assess the reliability of positive predictions, while recall or sensitivity measured the model’s ability to identify true positive cases. The F1 score was calculated as the harmonic mean of precision and recall to reflect the balance between the two measures. The area under the receiver operating characteristic curve (ROC AUC) was used to evaluate the ability of the model to distinguish between classes across different thresholds^34,35^. The area under the precision recall curve (PR AUC) was also calculated as it is particularly useful when class imbalance exists^36^. All metrics were calculated within each fold of Group K Fold cross-validation and the average across folds was used to summarize model performance^21^.

To understand which features contributed most to the classification results, we used SHapley Additive exPlanations (SHAP) on tree based models including CatBoost, LightGBM, XGBoost, and Histogram Gradient Boosting^37^. SHAP was applied both on data without imputation, taking advantage of the ability of tree based models to handle missing values^23,38^, and on imputed data to compare how feature importance might change after imputation. To avoid leakage, SHAP values were computed on validation folds using models trained only on their corresponding training folds, then aggregated across folds. SHAP values helped to identify how individual features influenced both individual predictions and the overall model outputs. We also used Local Interpretable Model Agnostic Explanations (LIME) to explain models trained on imputed datasets^39^. LIME was applied only after imputation since it requires complete data without missing values^40^. LIME was used to examine the local impact of features on predictions for samples in the test sets across cross-validation folds. Feature importance scores from LIME were averaged across samples and folds to identify consistently important predictors for each pairwise visit comparison.

## Results

### Demographic and clinical characteristics

An overview of patient demographics, sample sizes across years, hospitalization status and pre-existing comorbidities are shown in Table 1. In brief, the study cohort comprised 93 participants (60 female, 33 male) at baseline (visit 1) with a mean age of 48.9 ± 14.0 years. 34 (37%) patients had been hospitalized during acute COVID-19. Body mass index (BMI) was in the overweight range (26.1 ± 4.8 kg/m²). The mean time since infection was 6.2 ± 3.7 months at baseline visit 1 and 37.6 ± 5.1 months at visit 4 with 54 participants completing the final visit. Reasons for withdrawal were various and included unresponsiveness (*n* = 20), recovery (*n* = 3), death (*n* = 1), pregnancy (*n* = 1), illness (*n* = 6) and other reasons (*n* = 13). The distribution of cognitive and psychiatric test scores is presented in Table 2. Overall, participants showed largely normal global cognitive performance, poor sleep quality, severe fatigue, no clinically relevant anxiety or depressive symptoms, and no indications of excessive daytime sleepiness across visits.

**Table 1:** Sociodemographic and clinical parameters across study visits.

Parameter	Visit 1	*N*	Visit 2	*N*	Visit 3	*N*	Visit 4	*N*
Sex (female/male)	60/33		49/24		42/21		34/20	
Age (years)	48.9 ± 14.0	93	49.4 ± 12.5	73	50.2 ± 11.1	63	52.2 ± 11.6	54
Time since infection (months)	6.2 ± 3.7	93	14.0 ± 3.7	73	23.2 ± 4.3	62	37.6 ± 5.1	54
BMI (kg/m^2^)	26.1 ± 4.8	83	NA		27.0 ± 5.7	62	27.1 ± 5.7	53
Hospitalization during COVID-19	37.6%	34	30.1%	22	28.6%	18	33.3%	18
Intermediate/standard care	20.4%	19	13.7%	10	9.5%	6	11.1%	6
Intensive care	16.1%	15	16.4%	12	19.0%	12	22.2%	12
Cardiovaskular riskfaktors	40.9%	38	39.7%	29	38.1%	24	40.7%	22
Arterial hypertension	25.8%	24	27.4%	20	22.2%	14	25.9%	14
Diabetes	9.7%	9	9.6%	7	11.1%	7	11.1%	6
Dyslipidemia	11.8%	11	11.0%	8	7.9%	5	11.1%	6
Overweight	22.6%	21	21.9%	16	22.2%	14	20.4%	11
Neurological comorbidities	29.0%	27	28.8%	21	28.6%	18	27.8%	15
Stroke	2.2%	2	1.4%	1	0.0%	0	0.0%	0
TIA	2.2%	2	1.4%	1	1.6%	1	1.9%	1
Head trauma	3.2%	3	4.1%	3	4.8%	3	3.7%	2
Migraine	12.9%	12	12.3%	9	12.7%	8	11.1%	6
Epilepsy	2.2%	2	1.4%	1	1.6%	1	1.9%	1
Brain tumor	3.2%	3	4.1%	3	3.2%	2	3.7%	2
PNP	2.2%	2	0.0%	0	0.0%	0	0.0%	0
Movement disorder	2.2%	2	1.4%	1	1.6%	1	1.9%	1
Cognitive impairment	2.2%	2	1.4%	1	0.0%	0	1.9%	1
Psychiatric comorbidities	14.0%	13	13.7%	10	14.3%	9	14.8%	8
Depression	10.8%	10	11.0%	8	11.1%	7	11.1%	6
Anxiety disorder	2.2%	2	1.4%	1	1.6%	1	1.9%	1
Burn out	3.2%	3	4.1%	3	4.8%	3	5.6%	3
PTSD	2.2%	2	1.4%	1	1.6%	1	0.0%	0
Anorexia	2.2%	2	0.0%	0	0.0%	0	0.0%	0
Thyroid disease	23.7%	22	24.7%	18	22.2%	14	27.8%	15
Neurodermitis	3.2%	3	4.1%	3	3.2%	2	3.7%	2
Bronchial asthma	9.7%	9	9.6%	7	9.5%	6	11.1%	6
Pre-existing cardiac disease	18.3%	17	17.8%	13	14.3%	9	14.8%	8
COPD	4.3%	4	2.7%	2	3.2%	2	1.9%	1
History of cancer	6.5%	6	6.8%	5	6.3%	4	5.6%	3
Ulcerative colitis	2.2%	2	0.0%	0	0.0%	0	0.0%	0
Chron’s disease	2.2%	2	1.4%	1	1.6%	1	1.9%	1
Kidney disease	5.4%	5	5.5%	4	3.2%	2	3.7%	2
Liver disease	6.5%	6	5.5%	4	7.9%	5	9.3%	5

Data is given as mean with standard deviation (± SD) for age, time since infection and BMI, and as counts and percentages for the other variables. NA = Not available. Abbreviations: Transient Ischemic Attack (TIA), Polyneuropathy (PNP), Post-traumatic stress disorder (PTSD), Chronic Obstructive Pulmonary Disease (COPD).

**Table 2:** Cognitive and psychiatric test scores.

Parameter	Visit 1	*N*	Visit 2	*N*	Visit 3	*N*	Visit 4	*N*
MoCA	26.059 ± 3.275	85	27.159 ± 2.559	69	25.746 ± 3.188	63	26.481 ± 2.455	54
PSQI	9.509 ± 4.022	55	NA		9.440 ± 4.426	25	8.229 ± 3.549	35
FSMC cognitive	32.250 ± 10.477	72	33.300 ± 10.805	60	31.561 ± 12.282	57	31.075 ± 11.997	53
FSMC motor	30.958 ± 9.613	71	32.922 ± 9.724	64	32.167 ± 12.102	60	31.593 ± 12.217	54
FSMC total	63.296 ± 19.209	71	66.350 ± 19.445	60	63.386 ± 23.363	57	63.019 ± 23.314	53
HADS anxiety	6.902 ± 4.250	82	6.179 ± 4.119	67	6.383 ± 3.800	60	5.113 ± 3.550	53
HADS depression	5.222 ± 3.801	81	5.597 ± 4.445	67	5.607 ± 4.436	61	4.963 ± 3.469	54
HADS total	12.173 ± 7.225	81	11.776 ± 8.116	67	11.850 ± 7.353	60	10.075 ± 6.474	53
ESS	8.677 ± 5.283	65	9.714 ± 4.801	70	9.193 ± 5.142	57	8.685 ± 4.596	54
Digit span backward	8.959 ± 2.446	74	9.000 ± 2.236	73	9.238 ± 2.493	63	9.278 ± 2.351	54
Digit span forward	9.419 ± 2.305	74	9.836 ± 2.230	73	9.619 ± 2.113	63	10.000 ± 2.110	54
Verbal fluency phonetic	13.603 ± 4.530	73	11.639 ± 4.403	72	12.524 ± 3.737	63	12.189 ± 3.908	53
Verbal fluency semantic	21.753 ± 5.030	73	23.042 ± 5.226	72	15.762 ± 3.463	63	15.547 ± 3.232	53
SDMT	50.850 ± 11.602	60	49.861 ± 14.197	72	52.452 ± 13.816	62	52.623 ± 13.083	53
Stroop Interference	86.312 ± 23.984	64	88.273 ± 33.605	66	87.567 ± 27.658	60	82.170 ± 23.125	53
TAP alertness phasic (ms)	288.372 ± 78.982	86	281.863 ± 86.222	73	286.258 ± 113.102	62	277.415 ± 106.594	53
TAP alertness tonic (ms)	298.267 ± 94.549	86	304.986 ± 102.218	73	299.806 ± 110.457	62	274.283 ± 110.385	53
TAP divided attention auditory (ms)	641.508 ± 132.323	61	653.985 ± 129.017	67	672.867 ± 170.646	60	645.404 ± 115.756	52
TAP divided attention visual (ms)	803.295 ± 154.604	61	814.448 ± 149.236	67	757.567 ± 190.448	60	791.077 ± 120.751	52
TMT A (sec)	31.857 ± 13.197	91	32.973 ± 23.212	73	28.306 ± 10.800	62	27.685 ± 12.322	54
TMT B (sec)	81.484 ± 52.327	91	72.151 ± 48.554	73	63.790 ± 30.242	62	67.278 ± 39.485	54
VLMT trial 1	6.236 ± 2.236	72	7.219 ± 2.567	73	6.810 ± 2.764	63	6.792 ± 2.699	53
VLMT trial 5	12.847 ± 2.005	72	13.137 ± 2.388	73	12.667 ± 2.369	63	12.491 ± 2.926	53
VLMT delayed recall	10.625 ± 3.554	72	11.671 ± 3.508	73	10.762 ± 3.518	63	11.000 ± 3.563	53
VLMT recognition	13.667 ± 2.307	63	13.899 ± 1.816	69	13.317 ± 2.062	63	13.642 ± 1.952	53

Data is given as mean with standard deviation (SD). NA = Not available. Cut-off values: MoCA: < 26 impaired global cognition, ≥ 26 normal global cognition. PSQI: ≤ 5 good sleepers, > 5 poor sleepers. FSMC cognitive score: ≥22 mild fatigue, ≥ 28 moderate fatigue, ≥ 34 severe fatigue. FSMC motor score: ≥22 mild fatigue, ≥ 27 moderate fatigue, ≥ 32 severe fatigue. FSMC total score: ≥43 mild fatigue, ≥ 53 moderate fatigue, ≥ 63 severe fatigue. HADS-D anxiety score: ≤7 normal, 8–10 slightly increased. HADS-D depression score: ≤7 normal, 8–10 slightly increased. HADS-D total score: ≤14 normal, 15–21 questionable, > 21 increased. ESS: ≤10 average daytime sleepiness. >10 excessive daytime sleepiness. For neuropsychological variables, raw data are reported here.

### Classification performance across follow-up stages

Classification performance was evaluated across six pairwise visit comparisons using both original and imputed datasets. Results across models showed that classification performance was highest in comparisons involving longer follow-up intervals, especially visit1_vs_visit4 and visit2_vs_visit4, where accuracy values exceeded 90% and area under the ROC curve (ROC AUC) values reached 95% or higher. As the longitudinal trajectory progressed across visits, performance generally improved over time, with visit1_vs_visit4 and visit2_vs_visit4 showing the strongest results. Visit3_vs_ visit4 comparisons yielded comparatively lower performance, with accuracy typically between ~ 85% and 90%. To validate that clinical features captured genuine biological changes beyond temporal progression, we implemented a time-only baseline model achieving 100% accuracy (ROC-AUC = 1.00), confirming perfect temporal separability. Importantly, clinical feature models achieved 85–96% accuracy (ROC-AUC = 0.92–0.98), demonstrating that neuropsychological, immunological, and biomarker features independently distinguish disease stages with robust performance.

**Fig. 1:**
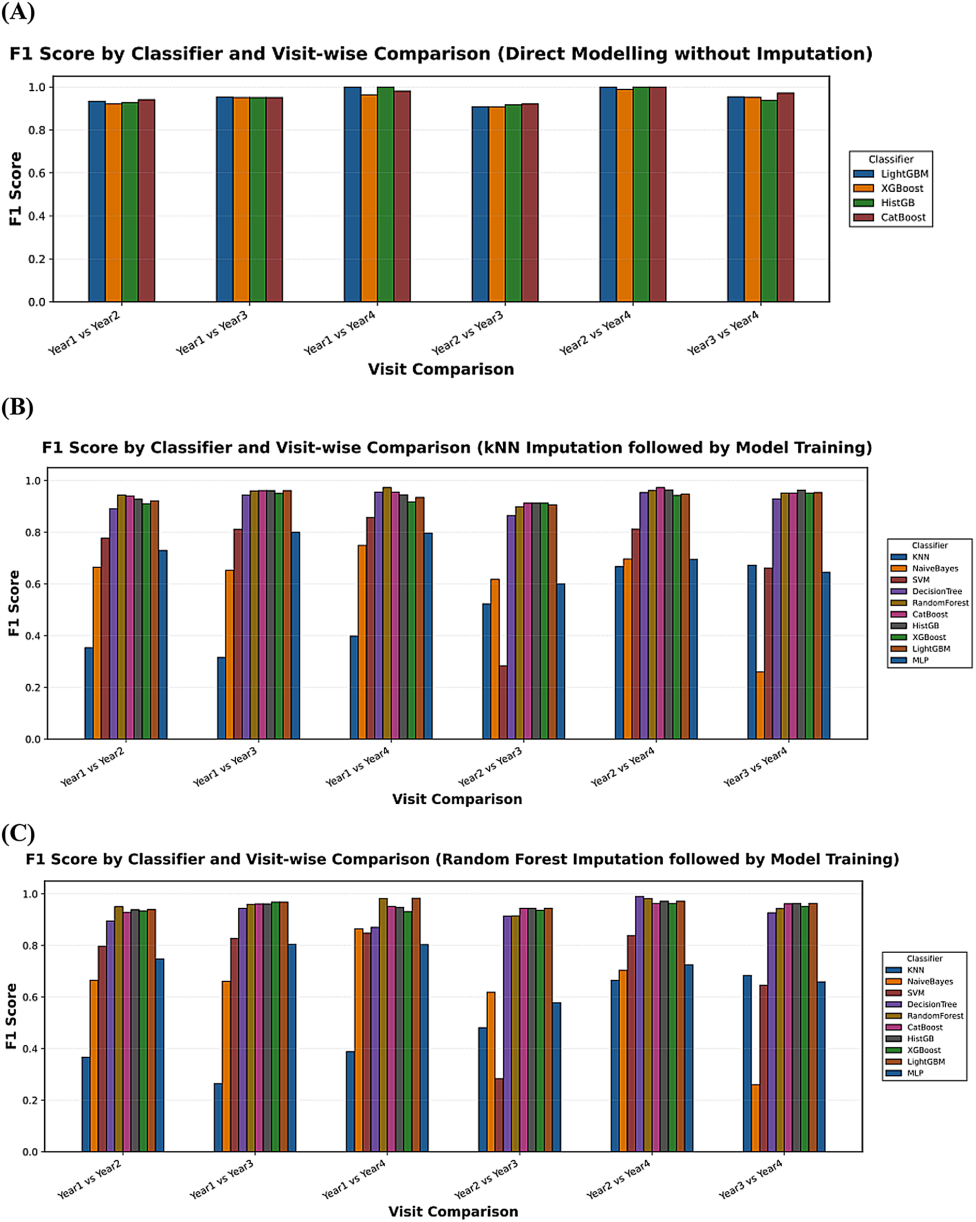
Longitudinal classification performance measured by F1 score for classifying the follow-up stage of patient status using multiple machine learning classifiers. Models were evaluated under three missing data handling strategies: (**A**) direct classification without imputation, preserving raw missingness; (**B**) classification after K-Nearest Neighbors (KNN) imputation; and (**C**) classification after Random Forest-based imputation.

Tree-based ensemble models, including CatBoost, LightGBM, XGBoost, and Histogram Gradient Boosting, consistently outperformed classical classifiers across all settings. CatBoost showed the highest and most stable accuracy across nearly all visit comparisons, especially in non-imputed data and imputed datasets using both K-nearest neighbors (KNN) and Random Forest (RF) imputation. Classical classifiers, such as Support Vector Machines, Naïve Bayes, Decision Tree, and Multi-Layer Perceptron, showed noticeably lower accuracy, particularly in the later visit wise comparisons. A summary of classification performance across all models and visit comparisons is presented in Fig. 1 and Supplemental Fig. 1.

### Key predictive features identified by explainability analyses

**Fig. 2:**
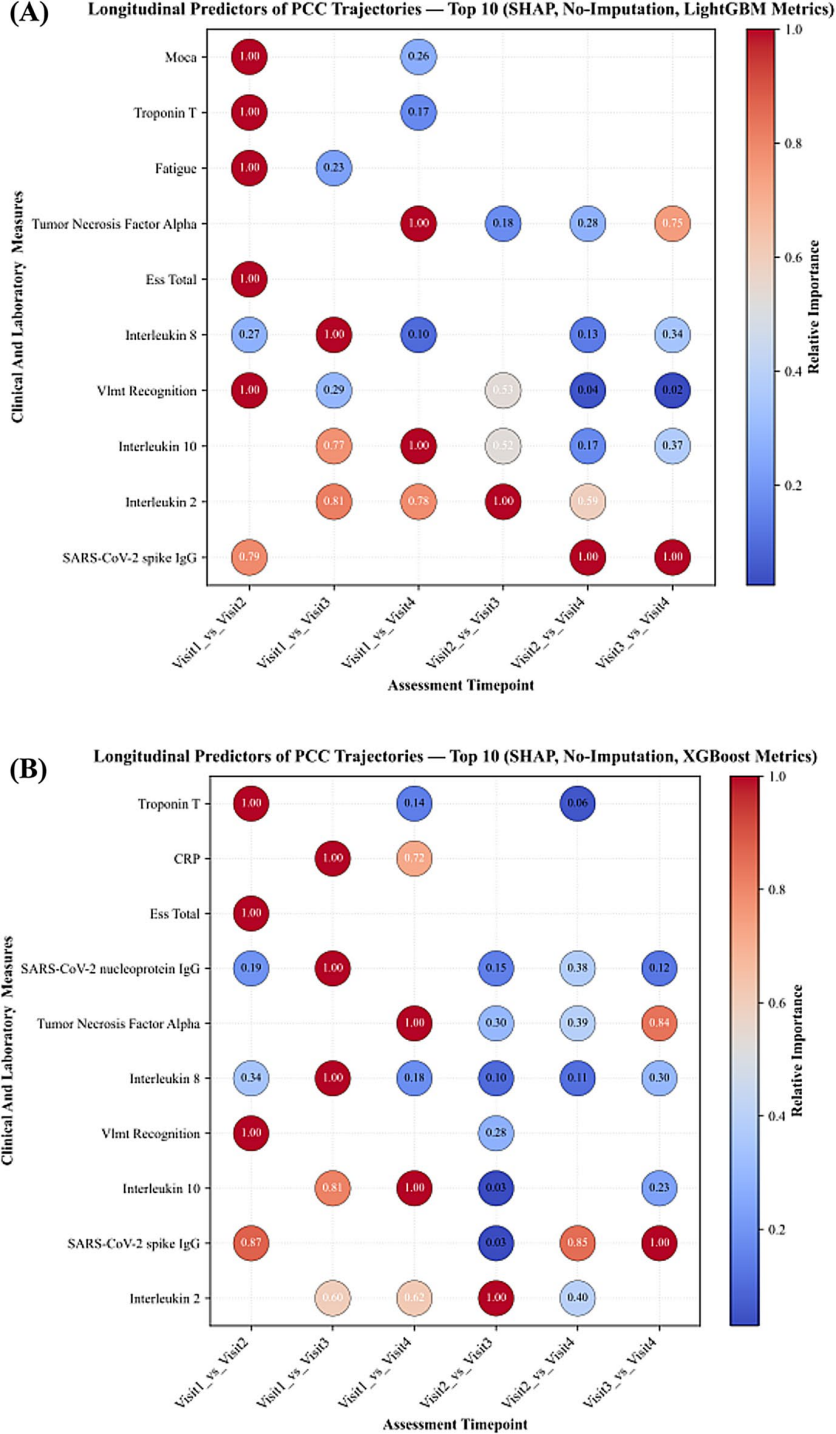
SHAP-based longitudinal feature importance for classifying the follow-up stage of patient status, computed using gradient boosting classifiers LightGBM and XGBoost trained without imputation through direct modeling on raw data.

Feature importance analysis using SHAP and LIME methods revealed a set of dominant predictors consistently contributing to model performance across all datasets. SARS-CoV-2 spike protein antibody levels, VLMT recognition, and inflammatory markers emerged as the most frequently identified and influential features in distinguishing between visits. SHAP analyses on non-imputed datasets consistently ranked SARS-CoV-2 antibody levels, VLMT recognition, and ESS total score as top predictors, alongside inflammatory markers, especially interleukins (IL) including IL-2, IL-8 and IL-10 were repeatedly identified across early and later visits, while IL-6 appeared only sporadically. Neuropsychological measures, particularly semantic fluency, were consistently observed in early visit comparisons, and fatigue emerged in some early visit contrasts but was not a stable predictor across imputations or later visits. Neuroinflammatory biomarkers emerged as prominent predictors in later visit comparisons. Figure 2 demonstrates the SHAP feature importance distribution in non-imputed data, highlighting the strength of these predictors.

**Fig. 3:**
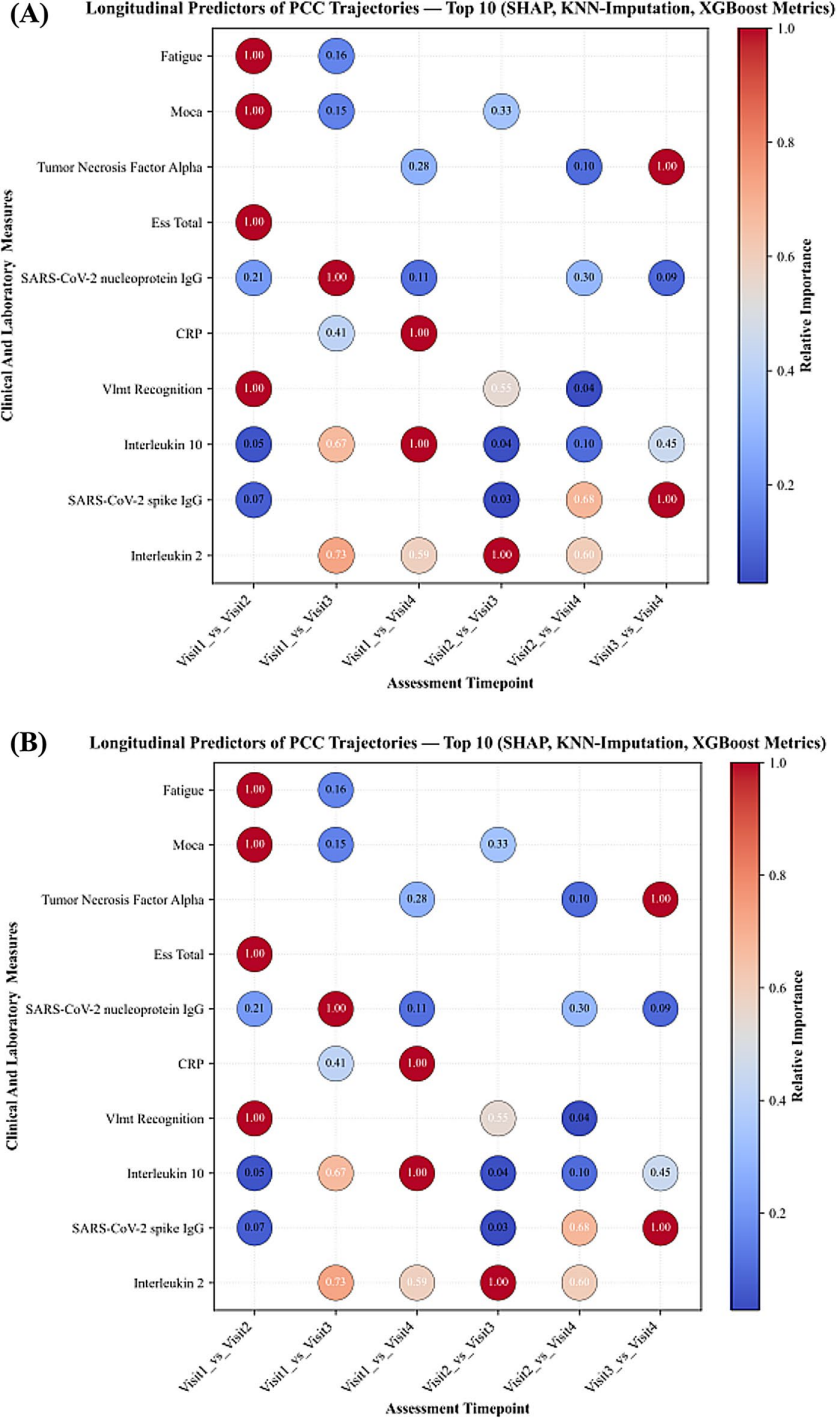
SHAP-based longitudinal feature importance for classifying the follow-up stage of patient status, computed using gradient boosting classifiers LightGBM and XGBoost after K-Nearest Neighbors (KNN) imputation of missing data.

**Fig. 4:**
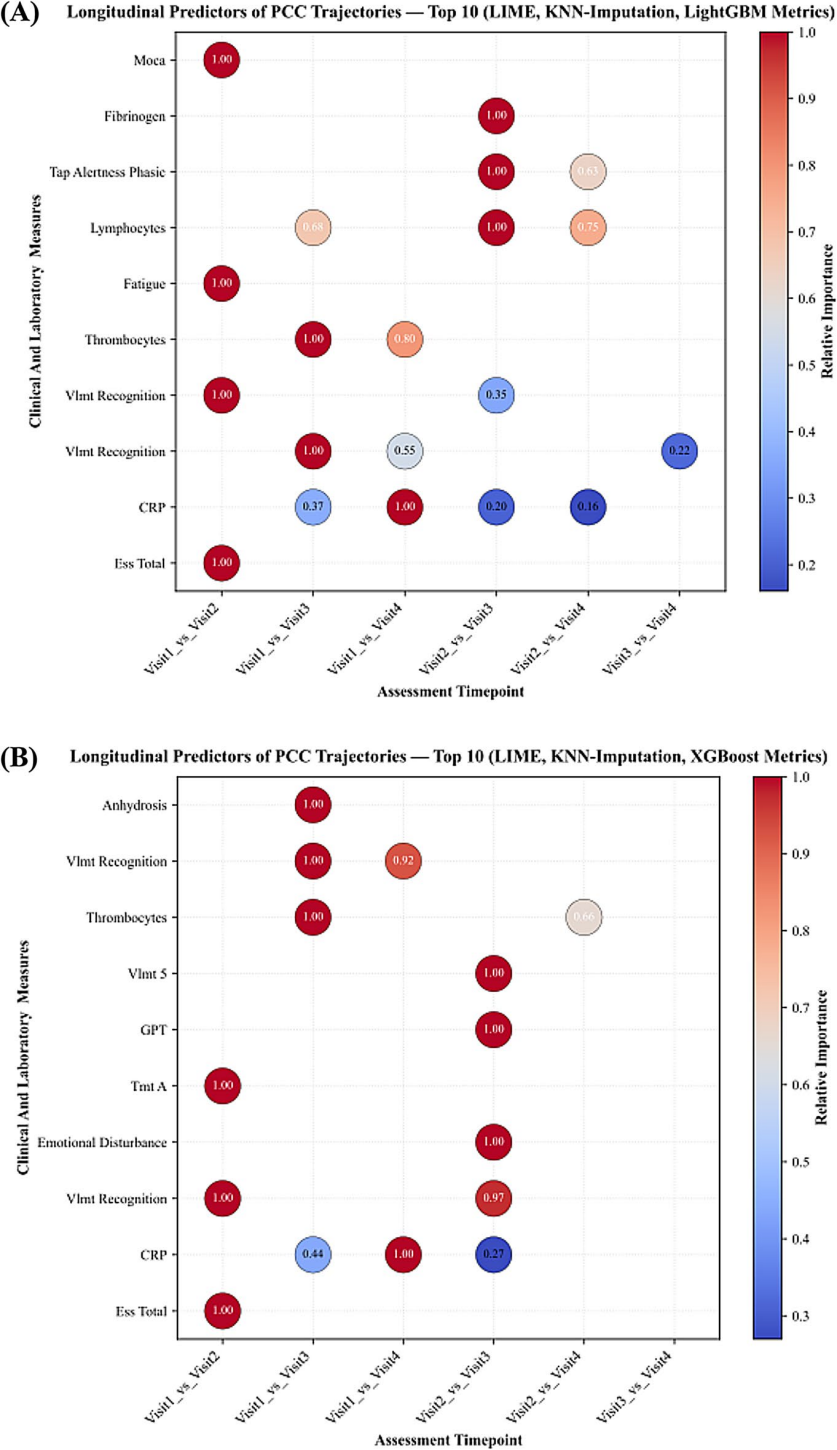
LIME-based longitudinal feature importance for classifying the follow-up stage of patient status, computed using gradient boosting classifiers LightGBM and XGBoost after K-Nearest Neighbors (KNN) imputation of missing data.

Following KNN imputation, SHAP analyses again confirmed SARS-CoV-2 antibodies, semantic fluency, and interleukins (IL-2, IL-8, IL-10) as dominant predictors. C-reactive Protein (CRP) and memory-recall measures appeared only occasionally, while IL-6 showed limited influence. Figure 3 demonstrates the SHAP feature importance distribution in KNN-imputed data, underscoring the persistence of these predictors across visits. In KNN-imputed datasets, LIME analyses also identified monocyte counts, semantic fluency, and SARS-CoV-2 antibodies as leading predictors, alongside symptom-related variables, such as smell disturbances and sleep disturbances, which were frequently ranked in early and mid-visit contrasts (Fig. 4).

In RF-imputed datasets, LIME analyses once more confirmed lymphocyte and monocyte counts, semantic fluency, and SARS-CoV-2 antibodies as top-ranking predictors. LIME also highlighted symptom-level variables (e.g., sleep disturbance, SDMT), which were more prominent in later visit contrasts. LIME feature importance rankings are visualized in Supplemental Fig. 2, while SHAP feature distributions after RF imputation are presented in Supplemental Fig. 3. The most frequently identified features across all analyses are summarized in Supplemental Tables 3–6, underscoring the consistent contributions from immune-inflammatory markers (IL-2, IL-8, IL-10, monocytes, lymphocytes), SARS-CoV-2 antibody responses and cognitive measures (VLMT recognition, semantic fluency, MoCA) along with symptom-level features highlighted by LIME analyses.

### Evolution of predictive patterns over time

Across visits, SARS-CoV-2 antibody measures and immune-inflammatory markers consistently ranked among the top predictor in all comparisons. In early visit contrasts (visit1_vs_visit2 and visit1_vs_visit3), neuropsychological measures such as semantic fluency appeared more frequently among the highest-ranked features, together with SARS-CoV-2 antibody measures. In later visit contrasts (visit2_vs_visit4 and visit1_vs_visit4), immune markers such IL-2, IL-8 and IL-10 were more prominent. IL-6 appeared occasionally in later comparisons, while fatigue was observed in early contrasts only. Classification performance followed a similar trajectory: accuracy was highest for longer-interval comparisons (visit1_vs_visit4 and visit2_vs_visit4), remained high for visit1_vs_visit2 and visit1_vs_visit3, and was lowest for visit3_vs_visit4.

## Discussion

In this three-year longitudinal study we employed advanced machine learning approaches and identified SARS-CoV-2 antibody levels, inflammatory and immune markers, and neuropsychological measures as the principal determinants of PCC, identifying differences across follow-up stages. While many studies have focused on short-term prognosis or single symptom domains, few have investigated how PCC trajectories unfold across multiple years using integrative clinical, laboratory, and neuropsychiatric data. This study contributes to filling that gap by applying machine learning techniques to a three-year longitudinal cohort of post-COVID patients, aiming to model the progression of PCC and identify key predictors of health status across different stages of recovery.

The use of machine learning in COVID-19 research has expanded rapidly over the past three years, with studies demonstrating its capacity to identify diagnostic markers, stratify disease severity, and predict hospital outcomes^7–9^. However, most models have been limited by short follow-up durations and relatively narrow feature sets. Recent work has emphasized the importance of moving beyond acute-phase predictions to understand the chronic manifestations of the disease^41^. For example, Groff et al. highlighted the long-term persistence of symptoms in a significant proportion of individuals, urging the need for models that can handle complex and heterogeneous data over time^42^. Our findings build on this imperative by leveraging repeated assessments of clinical, neuropsychiatric, and fluid markers, providing a more holistic view of PCC evolution over time.

Inflammatory markers such as IL-2, IL-10 and IL-8 appeared frequently among top-ranked features, which is not surprising given that the excessive production of pro-inflammatory cytokines is part of the acute COVID-19 pathology, and that altered cytokine profiles have consistently been reported in PCC^43,44^. One study investigating proteomic signatures in patients with post-acute sequelae after COVID-19, found persistent serum protein signatures in nearly 60% of study participants pointing to a an inflammatory subcategory of PCC^45^. However, cytokines are a heterogeneous, pleiotropic group of immune mediators with pro- as well as anti-inflammatory effects, requiring careful interpretation and findings on their individual relevance are mixed. Previous studies have found that altered levels of the proinflammatory and neutrophil-recruiting IL-8 was associated with PCC and that IL-10 was found to be associated with severity and mortality for patients with acute or post-acute SARS-CoV-2 infection^46,47^. Other studies in contrast, emphasized the importance of Il-6, which only sporadically appeared as a distinguishing feature in our analysis, to be associated with PCC and acting as a potential mediator of long-term neuropsychiatric symptoms of COVID-19^48,49^. Although findings regarding the relevance of distinct cytokines may differ, our findings support the role of an inflammatory driven pathogenesis in PCC. Further, we found that monocyte and lymphocyte counts emerged as a distinguishing feature in PCC trajectories, also pointing to an inflammatory subtype. This is in line with recent evidence that PCC monocyte percentage predicts subjective fatigue^50^ and manifests with T cell dysregulation, and uncoordinated adaptive immune response^51^. Taken together, multiplex immune-based panels maybe helpful to identify an inflammatory PCC subtype and guide future targeted therapy, given the limited efficacy of current empiric treatments^52,53^.

The presence of these variables across both SHAP and LIME analyses reinforces their stability and relevance, irrespective of the modeling approach or imputation strategy^8^. SARS-CoV-2 antibody levels against the spike protein was another key informative feature for distinguishing among follow-up years. The increase in antibodies against the spike protein in comparison to the relatively stable antibody levels against the nucleoprotein, indicate the availability of vaccines against SARS-CoV-2 during the course of the study. Although there is no reliable data for a therapeutic effect of vaccination in PCC, the protective role of SARS-CoV-2 vaccination has frequently been reported. A recent systematic review and meta-analysis demonstrated that SARS-CoV-2 vaccination is associated with a significantly reduced risk of developing PCC compared to no vaccination^54^. Thus, vaccination against SARS-CoV-2 is a key strategy to improve COVID-19 associated health outcomes.

During early follow-up, semantic fluency and fatigue contributed substantially to classification, highlighting the importance of early neuropsychological and fatigue screening in PCC. While semantic fluency performance largely fell within normative reference ranges and represents a relatively nonspecific diagnostic measure, the findings indicate that neuropsychiatric symptoms, despite their subjective and variable nature, convey critical information for characterizing recovery trajectories. Earlier studies have reported persistent cognitive dysfunction including impairments in logical reasoning, executive functions, and verbal memory, as some of the most debilitating symptoms of PCC, with varying timelines of improvement^55,56^. Our results support this view and extend it by quantifying the evolving importance of neuropsychological markers across different time intervals, showing how their predictive strength may rise or decline depending on the recovery phase.

Interestingly, the overall classification performance varied by the temporal gap between year pairs. Comparisons between more distant years (visit 1 vs. visit 4) yielded higher predictive accuracy, while adjacent year comparisons (visit 2 vs. visit 3) showed reduced separability. This indicates that changes in patient health status occur gradually, and the further apart the visits are, the more distinct the clinical profiles become. These findings are compatible with those of Groff et al., who observed that symptom improvement occurs incrementally in many patients, and that significant clinical shifts may only become evident after extended follow-up^42^.

The use of multiple imputation methods and modeling strategies in our study offered insights into the stability and reliability of machine learning classifiers in real-world clinical data settings. Although tree-based models like CatBoost and LightGBM performed well even without imputation, the inclusion of KNN and random forest imputed datasets allowed for broader classifier application and interpretability^57^. The consistent emergence of key variables across imputed and non-imputed analyses adds confidence to our results, while also emphasizing the value of missingness-aware modeling in longitudinal clinical research^57^. Importantly, the use of SHAP and LIME provided converging evidence of variable importance, bridging the gap between prediction and clinical interpretability an aspect critical for model translation into practice^5,8^.

The strengths of our study include a multimodal dataset spanning clinical assessments, neuropsychiatric testing, and fluid biomarkers together with an extended three-year follow-up period, enabling comprehensive phenotyping and cross-validation of findings across modalities. Nevertheless, the study has limitations that must be acknowledged. The cohort size was limited, reducing the representativeness of some subgroup analyses and increasing the variability of model estimates. The relatively small sample size per year also limits broader generalization, although careful cross-validation and group splitting were used to preserve longitudinal structure. Another limitation is the absence of external validation, which will be necessary in future work to confirm the stability and transportability of our models. Additionally, the absence of granular treatment histories, vaccination records, and lifestyle variables limits the explanatory scope of the models, as such factors may influence long-term outcomes. The interpretability methods used, though informative, also depend on underlying model architecture and assumptions, and their outputs should be viewed as suggestive rather than definitive. Lastly, discrepancies between subjective and objective cognitive deficits, previously linked to variations in inflammatory markers, represent a source of variance that the current machine learning model cannot capture, potentially leading to biased or distorted results^58,59^.

In conclusion, our study underscores that inflammatory and immune markers, SARS-CoV-2 antibody levels, and neuropsychological measures constitute principal determinants in PCC. These parameters should be systematically integrated into clinical and research frameworks to enhance diagnostic precision, patient stratification, and the development of targeted therapeutic interventions. Further, our study is one of the first detailed investigations of long-term PCC evolution using machine learning across a multi-domain, three-year cohort, showing that integration of predictive modeling with rigorous interpretability frameworks is suitable to identify potential, stable and dynamic biomarkers of recovery and symptom persistence. Future studies with larger and more diverse populations, coupled with richer longitudinal features, will be essential to translate these models into clinical practice.

## Supplementary Information

Below is the link to the electronic supplementary material.

Supplementary Material 1

## Data Availability

Deidentified clinical, neuropsychological and laboratory data are available on request from the corresponding author.
