# Supplementary material for: Longitudinal modeling of Post-COVID-19 condition over three years: A machine learning approach using clinical, neuropsychological, and fluid markers
Source: Sci Rep. 2026 Feb 14;16:6517. doi: 10.1038/s41598-026-37635-3 (PMC12909961; doi:10.1038/s41598-026-37635-3)
Supplement: Supplementary file 1 — Supplementary Material 1 [file 41598_2026_37635_MOESM1_ESM.docx]

**SUPPLEMENTAL DATA**

Table of Content

[Supplemental Table 1 Symptom prevalence across visits. 2](#_Toc212744928)

[Supplemental Table 2 Overview of all fluid biomarkers. 3](#_Toc212744929)

[Supplemental Table 3 Top 20 Informative Clinical Features Identified by SHAP and LIME across Machine Learning Models and Imputation Strategies. 6](#_Toc212744930)

[Supplemental Table 4. Classification Performance Metrics for Longitudinal COVID-19 Patient Visit Comparisons Using Direct Modelling without Imputation 10](#_Toc212744931)

[Supplemental Table 5. Classification Performance Metrics for Longitudinal COVID-19 Patient Visit Comparisons Using k-Nearest Neighbors Imputation followed by Model Training 11](#_Toc212744932)

[Supplemental Table 6. Classification Performance Metrics for Longitudinal COVID-19 Patient Visit Comparisons Using Random Forest Imputation followed by Model Training 12](#_Toc212744933)

[Supplemental Figure 1 ROC-AUC for classification performance across pairwise visit comparisons using machine learning models. 13](#_Toc212744934)

[Supplemental Figure 2 LIME based longitudinal feature importance for classifying the follow-up stage of patient status, computed using gradient boosting classifiers LightGBM and XGBoost after Random Forest-based imputation of missing data. 14](#_Toc212744935)

[Supplemental Figure 3 SHAP-based longitudinal feature importance for classifying the follow-up stage of patient status, computed using gradient boosting classifiers LightGBM and XGBoost after Random Forest-based imputation of missing data. 15](#_Toc212744936)

[References 16](#_Toc212744937)

#

# **Supplemental Table 1 Symptom prevalence across visits.** Overview of symptom prevalence and respective counts at each visit in post-COVID patients. Values are given as n (%). NA = not assessed.

| **Symptom, n (%)** | **Visit 1** | **Visit 2** | **Visit 3** | **Visit 4** |
| --- | --- | --- | --- | --- |
| Abdominal pain | 4 (4.4%) | 5 (6.8%) | 4 (6.3%) | 3 (5.7%) |
| Ageusia | 7 (7.7%) | 2 (2.7%) | 3 (4.8%) | 1 (1.9%) |
| Anosmia | 9 (9.9%) | 3 (4.1%) | 3 (4.8%) | 2 (3.8%) |
| Arthralgia | 13 (14.3%) | 9 (12.3%) | 13 (20.6%) | 10 (18.9%) |
| Attention/concentration difficulties | 56 (61.5%) | 53 (72.6%) | 45 (71.4%) | 38 (71.7%) |
| Chest pain | 6 (6.7%) | 4 (5.6%) | 4 (6.5%) | 2 (3.8%) |
| Coordination/balance difficulties | NA | 10 (13.9%) | 9 (14.3%) | 7 (13.2%) |
| Cough | 5 (5.5%) | 4 (5.5%) | 3 (4.8%) | 1 (1.9%) |
| Diarrhea | 4 (4.4%) | 5 (6.8%) | 2 (3.2%) | 2 (3.8%) |
| Dizziness | 14 (15.6%) | 4 (5.5%) | 3 (4.8%) | 2 (3.8%) |
| Dyspnea | 20 (22.0%) | 18 (24.7%) | 18 (28.6%) | 12 (22.6%) |
| Emotional disturbance | 18 (20.0%) | 16 (21.9%) | 14 (22.2%) | 9 (17.0%) |
| Excessive tiredness | 27 (30.0%) | 14 (19.2%) | 11 (17.5%) | 10 (18.9%) |
| Fever | 0 (0.0%) | 0 (0.0%) | 0 (0.0%) | 0 (0.0%) |
| General symptoms | 10 (11.0%) | 1 (1.4%) | 2 (3.2%) | 1 (1.9%) |
| Hair loss | 5 (5.6%) | 6 (8.3%) | 2 (3.2%) | 1 (1.9%) |
| Headache | 32 (35.6%) | 26 (35.6%) | 21 (33.3%) | 16 (30.2%) |
| Hearing problems | 8 (8.9%) | 8 (11.0%) | 6 (9.5%) | 3 (5.7%) |
| Hypogeusia | 25 (27.5%) | 18 (24.7%) | 15 (23.8%) | 11 (20.8%) |
| Hyposmia | 31 (34.1%) | 21 (28.8%) | 17 (27.0%) | 12 (22.6%) |
| Mouth ulcer | 0 (0.0%) | 1 (1.4%) | 1 (1.6%) | 0 (0.0%) |
| Muscle pain | 19 (21.1%) | 17 (23.3%) | 20 (31.7%) | 18 (34.0%) |
| Muscle weakness | 8 (8.9%) | 5 (6.8%) | 5 (7.9%) | 3 (5.7%) |
| Nasal problems | 1 (1.1%) | 2 (2.7%) | 2 (3.2%) | 0 (0.0%) |
| Nausea | 3 (3.3%) | 2 (2.7%) | 3 (4.8%) | 2 (3.8%) |
| Constipation | 3 (3.3%) | 7 (9.6%) | 5 (7.9%) | 6 (11.3%) |
| Orthostatic hypotension | 1 (1.1%) | 0 (0.0%) | 0 (0.0%) | 0 (0.0%) |
| Palpitations | 5 (5.6%) | 6 (8.2%) | 0 (0.0%) | 6 (11.3%) |
| Parageusia | 17 (18.7%) | 10 (13.7%) | 9 (14.3%) | 5 (9.4%) |
| Parasomnia | 5 (5.6%) | 2 (2.7%) | 1 (1.6%) | 1 (1.9%) |
| Parosmia | 16 (17.6%) | 19 (26.0%) | 14 (22.2%) | 9 (17.0%) |
| Phonophobia | 7 (7.8%) | 7 (9.6%) | 4 (6.3%) | 5 (9.4%) |
| Photophobia | 5 (5.6%) | 5 (6.8%) | 4 (6.3%) | 4 (7.5%) |
| Problems initiating sleep | 20 (22.2%) | 14 (19.2%) | 6 (9.8%) | 10 (19.2%) |
| Problems maintaining sleep | 27 (30.0%) | 27 (37.0%) | 13 (21.3%) | 20 (38.5%) |
| Reduced functional capacity | NA | 44 (60.3%) | 39 (61.9%) | 31 (58.5%) |
| Sensory disturbance/pain | 22 (23.7%) | 20 (27.4%) | 20 (31.7%) | 15 (28.3%) |
| Skin problems | 3 (3.4%) | 7 (9.6%) | 8 (12.7%) | 7 (13.2%) |
| Sleep problems | 35 (38.5%) | 27 (37.0%) | 24 (38.1%) | 21 (39.6%) |
| Smell disturbance | 40 (44.0%) | 12 (16.4%) | 14 (22.2%) | 8 (15.1%) |
| Sore throat | 3 (3.3%) | 3 (4.1%) | 2 (3.2%) | 1 (1.9%) |
| Taste disturbance | 37 (40.7%) | 21 (28.8%) | 18 (28.6%) | 13 (24.5%) |
| Temperature disturbance | 8 (8.8%) | 6 (8.2%) | 6 (9.5%) | 5 (9.4%) |
| Vertigo | 5 (5.6%) | 10 (13.7%) | 12 (19.0%) | 9 (17.0%) |
| Visual disturbance | 7 (7.8%) | 16 (21.9%) | 14 (22.2%) | 12 (22.6%) |
| Voice disturbance | 0 (0.0%) | 0 (0.0%) | 1 (1.6%) | 0 (0.0%) |
| Word-finding problems | 30 (33.0%) | 22 (30.1%) | 21 (33.3%) | 18 (34.0%) |

# **Supplemental Table 2 Overview of all fluid biomarkers.** Parameters grouped by category. Values are mean ± SD with sample size (N). NA = not available. *Based on published reference values according to mean cohort age.^1,2^

| **Parameter** | **Unit** | **Reference values** | **V1 Mean± SD** | **N** | **V2 Mean± SD** | **N** | **V3 Mean±SD** | **N** | **V4 Mean± SD** | **N** |
| --- | --- | --- | --- | --- | --- | --- | --- | --- | --- | --- |
| **Inflammatory and immune markers** | | | | | | | | | | |
| CRP | mg/l | <5 | 2.294 ± 3.534 | 47 | 3.472 ± 7.912 | 72 | 2.864 ± 4.928 | 62 | 2.458 ± 2.941 | 54 |
| Ferritin | ng/ml | 30.0 - 400.0 | 161.181 ± 325.235 | 60 | 124.701 ± 106.454 | 71 | 129.813 ± 140.303 | 63 | 123.222 ± 114.242 | 54 |
| Interleukin 10 | pg/ml | <9,1 | 1.474 ± 0.785 | 58 | 3.699 ± 16.567 | 70 | 3.285 ± 3.747 | 60 | 6.254 ± 9.488 | 47 |
| Interleukin 2 | pg/ml | <5,0 | 1.190 ± 0.726 | 56 | 1.141 ± 0.445 | 69 | 2.873 ± 2.336 | 60 | 2.600 ± 0.000 | 51 |
| Interleukin 6 | pg/ml | <7,0 | 2.151 ± 1.155 | 59 | 3.317 ± 3.687 | 71 | 3.303 ± 3.716 | 63 | 3.134 ± 2.672 | 53 |
| Interleukin 8 | pg/ml | <62 | 7.315 ± 7.918 | 58 | 14.406 ± 19.251 | 72 | 11.016 ± 7.573 | 62 | 14.795 ± 16.362 | 51 |
| Interleukin II receptor | U/ml | 223-710 | 284.925 ± 224.319 | 58 | 354.141 ± 125.910 | 71 | 353.873 ± 112.391 | 63 | 420.200 ± 174.351 | 54 |
| Procalcitonin | ng/ml | <0,5 | 0.028 ± 0.015 | 60 | 0.038 ± 0.030 | 71 | 0.040 ± 0.028 | 62 | 0.041 ± 0.045 | 54 |
| SARS Coronavirus 2 nucleoprotein IgG | AU/ml | negative <20, positive ≥24 | 57.417 ± 46.985 | 59 | 35.377 ± 37.963 | 72 | 39.081 ± 41.163 | 63 | 59.628 ± 42.009 | 54 |
| SARS Coronavirus 2 spike protein IgG | BAU/ ml | negative <33.8, positive ≥ 33.8 BAU/ml | 209.778 ± 162.224 | 13 | 341.909 ± 106.303 | 71 | 382.235 ± 239.340 | 63 | 1732.444 ± 517.415 | 54 |
| tumor necrosis factor alpha | pg/ml | <8,1 | 5.499 ± 3.945 | 60 | 8.202 ± 21.060 | 70 | 7.534 ± 10.469 | 62 | 9.041 ± 8.344 | 52 |
| **Liver and pancreas function tests** | | | | | | | | | | |
| GOT | U/l | < 50 | 22.297 ± 10.165 | 60 | 25.217 ± 10.112 | 71 | 24.047 ± 7.758 | 62 | 25.746 ± 9.754 | 54 |
| GPT | U/l | < 50 | 30.677 ± 30.246 | 60 | 26.276 ± 13.636 | 71 | 27.176 ± 13.951 | 62 | 28.544 ± 14.585 | 54 |
| LDH | U/l | <250 | 191.217 ± 38.120 | 60 | 201.451 ± 34.844 | 71 | 197.032 ± 34.551 | 62 | 195.130 ± 34.078 | 54 |
| albumin | g/dl | 3.5 - 5.2 | 4.704 ± 0.295 | 60 | 4.705 ± 0.255 | 71 | 4.646 ± 0.291 | 62 | 4.552 ± 0.281 | 54 |
| alkaline phosphatase | U/l | 40 - 130 | 72.183 ± 25.166 | 60 | 79.493 ± 50.325 | 71 | 76.242 ± 35.613 | 62 | 78.944 ± 28.536 | 54 |
| gamma-glutamyl transferase | U/l | < 60 | 41.067 ± 78.140 | 60 | 57.380 ± 245.478 | 71 | 47.339 ± 137.721 | 62 | 46.704 ± 73.405 | 54 |
| lipase | U/l | 13 - 60 | 35.002 ± 13.502 | 60 | 36.724 ± 16.150 | 71 | 38.961 ± 17.690 | 62 | 36.989 ± 11.442 | 54 |
| quick | % | 70 -120 | 106.407 ± 12.968 | 59 | 111.310 ± 15.290 | 71 | 108.698 ± 9.250 | 63 | 103.593 ± 14.097 | 54 |
| serum hemolysis index |  | - | 9.483 ± 3.383 | 58 | 10.338 ± 4.852 | 71 | 10.403 ± 4.833 | 62 | 11.962 ± 7.532 | 53 |
| serum icterus index |  | - | 1.034 ± 0.184 | 58 | 1.083 ± 0.366 | 72 | 1.097 ± 0.393 | 62 | 1.057 ± 0.305 | 53 |
| serum lipemia index |  | - | 11.276 ± 9.088 | 58 | 10.155 ± 7.187 | 71 | 10.177 ± 5.262 | 62 | 12.151 ± 12.558 | 53 |
| total bilirubin | mg/dl | <1.2 | 0.437 ± 0.192 | 60 | 0.514 ± 0.304 | 71 | 0.440 ± 0.226 | 62 | 0.494 ± 0.256 | 53 |
| **Renal and metabolic markers** | | | | | | | | | | |
| potassium | mmol/l | 3.5 - 5.1 | 4.374 ± 0.261 | 59 | 4.298 ± 0.362 | 71 | 4.284 ± 0.358 | 62 | 4.456 ± 0.566 | 54 |
| calcium corrected | mmol/l | 2.15 - 2.50 | 2.368 ± 0.088 | 60 | 2.387 ± 0.087 | 71 | 2.360 ± 0.097 | 62 | 2.243 ± 0.089 | 54 |
| creatinine | mg/dl | 0.70 - 1.20 | 0.777 ± 0.139 | 60 | 0.811 ± 0.182 | 71 | 0.847 ± 0.233 | 62 | 0.853 ± 0.218 | 54 |
| sodium | mmol/l | 136 - 145 | 139.973 ± 2.266 | 60 | 139.983 ± 1.924 | 71 | 140.658 ± 2.045 | 62 | 139.939 ± 1.867 | 54 |
| urea | mg/dl | 16.6 - 48.5 | 30.053 ± 7.842 | 60 | 30.161 ± 10.419 | 71 | 30.937 ± 13.674 | 62 | 33.389 ± 11.263 | 54 |
| urea/creatinine ratio |  | - | 30.053 ± 7.842 | 60 | 30.161 ± 10.419 | 71 | 31.860 ± 14.083 | 62 | 39.407 ± 9.952 | 54 |
| uric acid | mg/dl | 3.4 - 7.0 | 4.622 ± 1.134 | 60 | 4.775 ± 1.352 | 71 | 4.729 ± 1.359 | 62 | 4.675 ± 1.401 | 53 |
| **Lipid and glucose metabolism** | | | | | | | | | | |
| HDL cholesterol | mg/dl | > 35 | 63.288 ± 16.622 | 60 | 64.831 ± 18.091 | 71 | 64.713 ± 18.931 | 62 | 65.380 ± 20.031 | 54 |
| HbA1c | % | 4.0 - 6.0 | 5.168 ± 0.600 | 59 | 5.323 ± 0.922 | 71 | 5.459 ± 0.688 | 63 | 5.544 ± 0.766 | 52 |
| LDL cholesterol | mg/dl | < 100 mg/dl (optimal) | 120.282 ± 35.804 | 60 | 125.c75 ± 39.548 | 71 | 124.735 ± 38.237 | 62 | 127.083 ± 38.416 | 54 |
| cholesterol | mg/dl | < 200 | 194.352 ± 38.096 | 60 | 206.320 ± 45.836 | 71 | 206.216 ± 42.005 | 62 | 202.617 ± 39.837 | 54 |
| glucose | mg/dl | 74 - 106 | 88.358 ± 14.645 | 60 | 92.069 ± 19.402 | 71 | 98.108 ± 29.757 | 62 | 98.093 ± 18.988 | 54 |
| triglycerides | mg/dl | < 150 | 128.465 ± 82.541 | 60 | 148.939 ± 143.006 | 71 | 135.239 ± 71.300 | 62 | 133.494 ± 64.935 | 53 |
| **Hematology** | | | | | | | | | | |
| basophilic granulocytes | % | <1 | 0.606 ± 0.280 | 50 | 0.664 ± 0.293 | 70 | 0.623 ± 0.224 | 62 | 0.647 ± 0.250 | 53 |
| basophilic granulocytes absolute | /nl | < 0.08 | 0.039 ± 0.018 | 50 | 0.043 ± 0.018 | 70 | 0.044 ± 0.016 | 62 | 0.042 ± 0.015 | 53 |
| eosinophilic granulocytes | % | 1.0 - 7.0 | 1.742 ± 1.409 | 50 | 2.217 ± 1.750 | 70 | 1.976 ± 1.318 | 62 | 2.138 ± 1.573 | 53 |
| eosinophilic granulocytes absolute | /nl | 0.06 - 0.46 | 0.116 ± 0.106 | 50 | 0.143 ± 0.115 | 70 | 0.138 ± 0.100 | 62 | 0.141 ± 0.103 | 53 |
| erythroblasts | % |  | 0.000 ± 0.000 | 54 | 0.000 ± 0.000 | 71 | 0.000 ± 0.000 | 63 | 0.000 ± 0.000 | 54 |
| erythroblasts absolute | /nl |  | 0.000 ± 0.000 | 54 | 0.000 ± 0.000 | 71 | 0.000 ± 0.000 | 63 | 0.000 ± 0.000 | 54 |
| erythrocytes | /pl | 4.6 - 6.1 | 4.692 ± 0.400 | 54 | 4.709 ± 0.424 | 71 | 4.734 ± 0.443 | 63 | 4.797 ± 0.446 | 54 |
| hematocrit | % | 40.1 - 50.0 | 41.635 ± 3.058 | 54 | 41.854 ± 3.379 | 71 | 42.186 ± 3.529 | 63 | 42.176 ± 3.858 | 54 |
| hemoglobin | g/dl | 13.7 - 17.5 | 14.078 ± 1.264 | 54 | 14.163 ± 1.205 | 71 | 14.262 ± 1.246 | 63 | 14.417 ± 1.367 | 54 |
| immature granulocyte | % | 0 - 0.74 | 0.404 ± 0.405 | 50 | 0.317 ± 0.200 | 70 | 0.385 ± 0.206 | 62 | 0.387 ± 0.259 | 53 |
| leukocytes | /nl | 4.2 - 9.1 | 7.465 ± 5.098 | 54 | 6.801 ± 2.032 | 71 | 7.231 ± 1.858 | 63 | 6.686 ± 1.749 | 54 |
| lymphocytes | % | 22.0 - 53.0 | 29.024 ± 7.599 | 50 | 29.597 ± 7.188 | 70 | 29.524 ± 7.206 | 62 | 29.804 ± 7.976 | 53 |
| lymphocytes absolute | /nl | 1.0 - 2.9 | 1.869 ± 0.510 | 50 | 1.929 ± 0.537 | 70 | 2.069 ± 0.589 | 62 | 1.913 ± 0.575 | 53 |
| mean corpuscular hemoglobin | pg | 25.7 - 32.2 | 30.019 ± 1.386 | 54 | 30.118 ± 1.378 | 71 | 30.176 ± 1.444 | 63 | 30.089 ± 1.437 | 54 |
| mean corpuscular hemoglobin concentration | g/dl | 32.0 - 36.0 | 33.789 ± 1.074 | 54 | 33.835 ± 0.855 | 71 | 33.816 ± 1.148 | 63 | 34.193 ± 0.954 | 54 |
| mean corpuscular volume | fl | 79.0 - 92.2 | 88.861 ± 3.066 | 54 | 89.025 ± 3.584 | 71 | 89.267 ± 3.885 | 63 | 88.033 ± 4.081 | 54 |
| monocytes | % | 5.0 - 12.0 | 7.448 ± 1.907 | 50 | 7.997 ± 2.231 | 70 | 7.581 ± 2.056 | 62 | 7.611 ± 1.832 | 53 |
| monocytes absolute | /nl | 0.2 - 0.6 | 0.490 ± 0.175 | 50 | 0.523 ± 0.160 | 70 | 0.534 ± 0.176 | 62 | 0.499 ± 0.157 | 53 |
| red cell distribution width - coefficient of variation | % | 11.0 - 16.0 | 12.691 ± 0.759 | 54 | 12.794 ± 0.833 | 71 | 12.883 ± 0.900 | 63 | 12.948 ± 0.792 | 54 |
| segmented neutrophilic granulocytes | % | 34.0 - 68.0 | 60.776 ± 8.643 | 50 | 59.207 ± 8.032 | 70 | 59.911 ± 8.285 | 62 | 59.413 ± 8.591 | 53 |
| segmented neutrophilic granulocytes absolute | /nl | 1.6 - 7.1 | 4.198 ± 1.858 | 50 | 4.011 ± 1.534 | 70 | 4.323 ± 1.345 | 62 | 3.968 ± 1.338 | 53 |
| thrombocytes | /nl | 150 - 400 | 254.260 ± 56.626 | 50 | 254.857 ± 55.788 | 70 | 280.667 ± 74.306 | 63 | 261.593 ± 61.992 | 54 |
| **Coagulation** | | | | | | | | | | |
| INR | NA |  | 0.973 ± 0.085 | 59 | 0.950 ± 0.103 | 71 | 0.952 ± 0.052 | 63 | 1.004 ± 0.214 | 54 |
| aPTT | sec | 21 - 29 | 30.942 ± 4.203 | 59 | 31.227 ± 3.691 | 71 | 30.922 ± 3.349 | 63 | 30.994 ± 3.085 | 54 |
| antithrombin III | % | 80 - 120 | 106.847 ± 10.198 | 59 | 103.380 ± 9.459 | 71 | 102.143 ± 10.231 | 63 | 103.389 ± 11.148 | 54 |
| d-dimers | ug/l | <600 | 351.119 ± 442.249 | 59 | 330.070 ± 224.775 | 71 | 327.758 ± 219.140 | 62 | 333.537 ± 198.313 | 54 |
| fibrinogen | mg/dl | 170 - 420 | 289.831 ± 52.434 | 59 | 293.141 ± 70.581 | 71 | 307.429 ± 58.134 | 63 | 313.574 ± 54.789 | 54 |
| plasma thrombin time | sec | 16 - 19 | 16.137 ± 19.529 | 59 | 14.706 ± 7.599 | 71 | 14.068 ± 0.863 | 63 | 14.524 ± 0.885 | 54 |
| **Cardiac markers** | | | | | | | | | | |
| NT-proBNP | pg/ml | <210 | 77.361 ± 77.915 | 60 | 112.565 ± 232.131 | 71 | 79.607 ± 70.417 | 63 | 67.554 ± 55.775 | 54 |
| troponin T | pg/ml | < 14.0 | 7.116 ± 15.104 | 60 | 11.728 ± 41.065 | 71 | 6.797 ± 5.182 | 63 | 7.217 ± 6.042 | 54 |
| **Neuronal injury markers** | | | | | | | | | | |
| Gfap | pg/ml | <242* | 139.502 ± 314.541 | 71 | 111.186 ± 44.217 | 69 | 124.378 ± 52.297 | 62 | 139.104 ± 57.905 | 45 |
| Nfl | pg/ml | <15* | 9.692 ± 13.208 | 71 | 9.229 ± 5.656 | 69 | 10.242 ± 4.701 | 62 | 10.226 ± 4.882 | 45 |
| **Vitamins** | | | | | | | | | | |
| folic acid | ng/ml | 4.6 - 18.7 | 8.696 ± 4.573 | 59 | 9.828 ± 5.537 | 71 | 8.580 ± 4.610 | 63 | 8.827 ± 4.906 | 54 |
| vitamin B12 | pg/ml | 197 - 771 | 483.949 ± 250.174 | 59 | 484.535 ± 161.982 | 71 | 476.694 ± 143.888 | 62 | 487.926 ± 157.897 | 54 |
| **Thyroid parameters** | | | | | | | | | | |
| TSH | mU/l | 0.27 - 4.20 | 1.385 ± 0.737 | 60 | 1.533 ± 0.773 | 71 | 1.604 ± 1.008 | 63 | 1.733 ± 1.398 | 54 |

**Supplemental Table 3 Top 20 Informative Clinical Features Identified by SHAP and LIME across Machine Learning Models and Imputation Strategies.**

| **Imputation Method** | **Classifier** | **Feature** | **Mean Absolute Contribution** |
| --- | --- | --- | --- |
| **LIME Top Features** | | | |
| KNN Imputation | LightGBM | Fatigue | 0,820235 |
|  |  | Moca | 0,673415 |
|  |  | Vlmt Recognition | 0,668967 |
|  |  | Bowel Incontinency | 0,605276 |
|  |  | Attention Concentration Problems | 0,584526 |
|  |  | Voice Disturbance | 0,540606 |
|  |  | Lymphocytes | 0,492839 |
|  |  | Nasal Problems | 0,423331 |
|  |  | Vlmt 5 | 0,413069 |
|  |  | Urinary Incontinency | 0,384506 |
|  |  | Memory Impairement | 0,365965 |
|  |  | Monocytes | 0,348462 |
|  |  | Nausea | 0,345919 |
|  |  | Fibrinogen | 0,345446 |
|  |  | Muscle Pain | 0,342412 |
|  |  | Sore Throat | 0,327655 |
|  |  | Parasomnia | 0,306214 |
|  |  | Ageusia | 0,30454 |
|  |  | General Symptomps | 0,298601 |
|  |  | Cough | 0,290694 |
|  | XGBoost | Vlmt Recognition | 0,995907 |
|  |  | GPT | 0,591176 |
|  |  | Vlmt 5 | 0,58651 |
|  |  | Tmt A | 0,514492 |
|  |  | Bowel Incontinency | 0,375169 |
|  |  | Voice Disturbance | 0,370758 |
|  |  | Lymphocytes | 0,350084 |
|  |  | Nasal Problems | 0,346419 |
|  |  | Leukocytes | 0,333539 |
|  |  | Fibrinogen | 0,331366 |
|  |  | Fatigue | 0,318742 |
|  |  | Albumin | 0,30048 |
|  |  | Monocytes | 0,288181 |
|  |  | Taste Disturbance | 0,273633 |
|  |  | Digits Forward | 0,271208 |
|  |  | Urinary Incontinency | 0,267081 |
|  |  | Nausea | 0,260487 |
|  |  | Moca | 0,25594 |
|  |  | Emotional Disturbance | 0,246812 |
|  |  | Sore Throat | 0,245791 |
| RF Imputation | LightGBM | Fatigue | 0,907729 |
|  |  | Bowel Incontinency | 0,707207 |
|  |  | Vlmt Recognition | 0,684914 |
|  |  | Orthostetic Hypotension | 0,638382 |
|  |  | Tap Dual Visual | 0,546603 |
|  |  | Nasal Problems | 0,462551 |
|  |  | Attention Concentration Problems | 0,46027 |
|  |  | Voice Disturbance | 0,439054 |
|  |  | Mouth Ulcera | 0,430519 |
|  |  | Lymphocytes | 0,389689 |
|  |  | Monocytes | 0,358286 |
|  |  | Urinary Incontinency | 0,341249 |
|  |  | Nfl | 0,338162 |
|  |  | Memory Impairement | 0,336832 |
|  |  | Nausea | 0,320201 |
|  |  | Sore Throat | 0,314191 |
|  |  | Parasomnia | 0,29841 |
|  |  | Ageusia | 0,286056 |
|  |  | Moca | 0,285721 |
|  |  | Cough | 0,274816 |
|  | XGBoost | Vlmt Recognition | 0,814003 |
|  |  | Tmt A | 0,591421 |
|  |  | Bowel Incontinency | 0,469515 |
|  |  | Orthostetic Hypotension | 0,466229 |
|  |  | Lymphocytes | 0,379599 |
|  |  | Mouth Ulcera | 0,364222 |
|  |  | Tap Dual Visual | 0,360551 |
|  |  | Nasal Problems | 0,349912 |
|  |  | Nfl | 0,339342 |
|  |  | Moca | 0,32354 |
|  |  | Voice Disturbance | 0,303745 |
|  |  | Monocytes | 0,291893 |
|  |  | Urinary Incontinency | 0,281413 |
|  |  | Nausea | 0,255344 |
|  |  | Parasomnia | 0,245798 |
|  |  | Albumin | 0,243375 |
|  |  | Sore Throat | 0,241427 |
|  |  | Ageusia | 0,236651 |
|  |  | General Symptomps | 0,23498 |
|  |  | Fatigue | 0,230776 |
| **SHAP Top Features** | | | |
| Direct Modeling (No Imputation) | LightGBM | SARS-CoV-2 spike IgG | 1,825441 |
|  |  | Interleukin 2 | 1,70638 |
|  |  | Interleukin 10 | 0,697506 |
|  |  | Vlmt Recognition | 0,249679 |
|  |  | Fatigue | 0,169909 |
|  |  | Ess Total | 0,164859 |
|  |  | Tumor Necrosis Factor Alpha | 0,128699 |
|  |  | Vlmt 7 | 0,119521 |
|  |  | Moca | 0,114383 |
|  |  | CRP | 0,104404 |
|  |  | Interleukin 8 | 0,104318 |
|  |  | Thyroid Stimulating Hormone | 0,096013 |
|  |  | INR | 0,090389 |
|  |  | Triglycerides | 0,076304 |
|  |  | Stroop | 0,068514 |
|  |  | Troponin T | 0,06816 |
|  |  | Eosinophilic Granulocytes Absolute | 0,06009 |
|  |  | Fibrinogen | 0,058367 |
|  |  | SARS-CoV-2 nucleoprotein IgG | 0,055226 |
|  |  | Immature Granulocyte | 0,054445 |
|  | XGBoost | Interleukin 2 | 2,17655 |
|  |  | SARS-CoV-2 spike IgG | 2,045114 |
|  |  | Interleukin 10 | 0,598014 |
|  |  | Vlmt Recognition | 0,316044 |
|  |  | Interleukin 8 | 0,142404 |
|  |  | CRP | 0,140382 |
|  |  | Tumor Necrosis Factor Alpha | 0,127093 |
|  |  | SARS-CoV-2 nucleoprotein IgG | 0,103015 |
|  |  | Ess Total | 0,095449 |
|  |  | Lymphocytes Absolute | 0,086888 |
|  |  | Moca | 0,081325 |
|  |  | Troponin T | 0,070809 |
|  |  | Tmt A | 0,06958 |
|  |  | Digits Backward | 0,067307 |
|  |  | Thrombocytes | 0,064088 |
|  |  | Red Cell Distribution Width - Coefficient | 0,061896 |
|  |  | Gfap | 0,060009 |
|  |  | D-dimers | 0,059988 |
|  |  | Nfl | 0,05957 |
|  |  | Fluency Semantic | 0,05234 |
| KNN Imputation | LightGBM | Interleukin 2 | 1,941688 |
|  |  | SARS-CoV-2 spike IgG | 1,100726 |
|  |  | Interleukin 10 | 0,780758 |
|  |  | Vlmt Recognition | 0,221507 |
|  |  | Fatigue | 0,187604 |
|  |  | Attention Concentration Problems | 0,143513 |
|  |  | Tumor Necrosis Factor Alpha | 0,086909 |
|  |  | Thyroid Stimulating Hormone | 0,086089 |
|  |  | Fibrinogen | 0,080322 |
|  |  | Memory Impairement | 0,077422 |
|  |  | SARS-CoV-2 nucleoprotein IgG | 0,076868 |
|  |  | Interleukin II Receptor | 0,075222 |
|  |  | Troponin T | 0,072008 |
|  |  | Tmt A | 0,070277 |
|  |  | Lymphocytes Absolute | 0,064965 |
|  |  | Eosinophilic Granulocytes | 0,061755 |
|  |  | Moca | 0,059969 |
|  |  | CRP | 0,057466 |
|  |  | GOT | 0,056833 |
|  |  | Creatine Cinase | 0,05444 |
|  | XGBoost | Interleukin 2 | 2,329879 |
|  |  | SARS-CoV-2 spike IgG | 1,235931 |
|  |  | Interleukin 10 | 0,611303 |
|  |  | Vlmt Recognition | 0,279816 |
|  |  | SARS-CoV-2 nucleoprotein IgG | 0,113558 |
|  |  | Moca | 0,099443 |
|  |  | Tmt A | 0,086791 |
|  |  | Fatigue | 0,079097 |
|  |  | Vlmt 5 | 0,07236 |
|  |  | Tumor Necrosis Factor Alpha | 0,068635 |
|  |  | Fsmc Motor | 0,06262 |
|  |  | Fibrinogen | 0,060528 |
|  |  | Total Bilirubin | 0,054739 |
|  |  | Creatine Cinase | 0,053637 |
|  |  | Red Cell Distribution Width - Coefficient | 0,053255 |
|  |  | Serum Hemolysis Index | 0,052637 |
|  |  | Lymphocytes Absolute | 0,052618 |
|  |  | Interleukin II Receptor | 0,050246 |
|  |  | Troponin T | 0,04722 |
|  |  | Interleukin 8 | 0,044678 |
| RF Imputation | LightGBM | Vlmt Recognition | 0,248077 |
|  |  | Attention Concentration Problems | 0,164948 |
|  |  | Fatigue | 0,164865 |
|  |  | Procalcitonin | 0,089458 |
|  |  | Nfl | 0,06839 |
|  |  | Vlmt 7 | 0,061605 |
|  |  | Hads Depression | 0,056852 |
|  |  | Vlmt 5 | 0,053276 |
|  |  | Fibrinogen | 0,049149 |
|  |  | Stroop | 0,034863 |
|  |  | Creatinine | 0,033958 |
|  |  | Tmt A | 0,032853 |
|  |  | Albumin | 0,030549 |
|  |  | Gfap | 0,030313 |
|  |  | HBA1L | 0,028968 |
|  |  | GPT | 0,027028 |
|  |  | GOT | 0,025197 |
|  |  | Moca | 0,024704 |
|  |  | Fluency Phonetic | 0,022683 |
|  |  | Fluency Semantic | 0,019933 |
|  | XGBoost | Interleukin 2 | 2,205348 |
|  |  | SARS-CoV-2 spike IgG | 1,452825 |
|  |  | Interleukin 10 | 0,575453 |
|  |  | Vlmt Recognition | 0,27681 |
|  |  | Tumor Necrosis Factor Alpha | 0,132488 |
|  |  | Interleukin 8 | 0,119295 |
|  |  | Vlmt 7 | 0,104621 |
|  |  | SARS-CoV-2 nucleoprotein IgG | 0,094565 |
|  |  | Troponin T | 0,088201 |
|  |  | Vlmt 5 | 0,078551 |
|  |  | Fibrinogen | 0,072668 |
|  |  | Procalcitonin | 0,071912 |
|  |  | Interleukin II Receptor | 0,061532 |
|  |  | Fatigue | 0,057713 |
|  |  | Lymphocytes Absolute | 0,057453 |
|  |  | Age | 0,056891 |
|  |  | Digits Backward | 0,056781 |
|  |  | Tmt A | 0,05538 |
|  |  | Segmented Neutrophilic Granulocytes Absolute | 0,051313 |
|  |  | Lymphocytes | 0,049569 |

# **Supplemental Table 4**. Classification Performance Metrics for Longitudinal COVID-19 Patient Visit Comparisons Using Direct Modelling without Imputation

| **Comparison** | **Model** | **AUC (95% CI)** | **Sensitivity** | **Specificity** | **Youden Index** |
| --- | --- | --- | --- | --- | --- |
| Year1_vs_Year2 | LightGBM | 0.987 (0.966-1.007) | 0.959 ± 0.033 | 0.452 ± 0.471 | 0.411 ± 0.498 |
|  | XGBoost | 0.977 (0.956-0.997) | 0.958 ± 0.057 | 0.561 ± 0.499 | 0.519 ± 0.537 |
|  | HistGB | 0.984 (0.965-1.002) | 0.946 ± 0.027 | 0.300 ± 0.401 | 0.246 ± 0.423 |
|  | CatBoost | 0.980 (0.953-1.007) | 0.972 ± 0.034 | 0.525 ± 0.523 | 0.498 ± 0.552 |
| Year2_vs_Year3 | LightGBM | 0.950 (0.887-1.013) | 0.936 ± 0.079 | 0.605 ± 0.466 | 0.541 ± 0.526 |
|  | XGBoost | 0.958 (0.911-1.006) | 0.922 ± 0.072 | 0.438 ± 0.475 | 0.360 ± 0.523 |
|  | HistGB | 0.954 (0.895-1.012) | 0.951 ± 0.066 | 0.605 ± 0.466 | 0.556 ± 0.518 |
|  | CatBoost | 0.968 (0.927-1.009) | 0.936 ± 0.079 | 0.605 ± 0.466 | 0.541 ± 0.526 |
| Year1_vs_Year3 | LightGBM | 0.996 (0.991-1.001) | 0.954 ± 0.062 | 0.654 ± 0.410 | 0.608 ± 0.447 |
|  | XGBoost | 0.994 (0.981-1.007) | 0.938 ± 0.090 | 0.759 ± 0.274 | 0.698 ± 0.337 |
|  | HistGB | 0.997 (0.991-1.002) | 0.938 ± 0.090 | 0.759 ± 0.274 | 0.698 ± 0.337 |
|  | CatBoost | 0.986 (0.971-1.002) | 0.938 ± 0.090 | 0.759 ± 0.274 | 0.698 ± 0.337 |
| Year2_vs_Year4 | LightGBM | 1.000 (1.000-1.000) | 1.000 ± 0.000 | 1.000 ± 0.000 | 1.000 ± 0.000 |
|  | XGBoost | 1.000 (1.000-1.000) | 0.980 ± 0.040 | 0.867 ± 0.207 | 0.847 ± 0.239 |
|  | HistGB | 1.000 (1.000-1.000) | 1.000 ± 0.000 | 1.000 ± 0.000 | 1.000 ± 0.000 |
|  | CatBoost | 1.000 (1.000-1.000) | 1.000 ± 0.000 | 1.000 ± 0.000 | 1.000 ± 0.000 |
| Year1_vs_Year4 | LightGBM | 1.000 (1.000-1.000) | 1.000 ± 0.000 | 1.000 ± 0.000 | 1.000 ± 0.000 |
|  | XGBoost | 0.999 (0.997-1.001) | 0.945 ± 0.045 | 0.668 ± 0.294 | 0.614 ± 0.332 |
|  | HistGB | 1.000 (1.000-1.000) | 1.000 ± 0.000 | 1.000 ± 0.000 | 1.000 ± 0.000 |
|  | CatBoost | 1.000 (1.000-1.000) | 0.964 ± 0.045 | 0.814 ± 0.204 | 0.777 ± 0.245 |
| Year3_vs_Year4 | LightGBM | 0.982 (0.951-1.013) | 0.962 ± 0.047 | 0.594 ± 0.490 | 0.556 ± 0.524 |
|  | XGBoost | 0.993 (0.983-1.003) | 0.925 ± 0.069 | 0.596 ± 0.365 | 0.522 ± 0.410 |
|  | HistGB | 0.974 (0.928-1.021) | 0.962 ± 0.047 | 0.594 ± 0.490 | 0.556 ± 0.524 |
|  | CatBoost | 0.988 (0.969-1.006) | 0.962 ± 0.047 | 0.654 ± 0.413 | 0.616 ± 0.451 |

Classification performance metrics showing area under the ROC curve (AUC) with 95% confidence intervals, sensitivity (true positive rate), specificity (true negative rate), and Youden Index (J = Sensitivity + Specificity - 1) for temporal disease progression prediction in COVID-19 patients. Values represent mean ± standard deviation across 5-fold cross-validation using gradient boosting ensemble methods. Perfect classification (AUC = 1.000, J = 1.000) was achieved for comparisons spanning longer temporal intervals (Year1 vs Year3/Year4, Year2 vs Year4, Year3 vs Year4), demonstrating clear disease trajectory separation over time.

# **Supplemental Table 5**. Classification Performance Metrics for Longitudinal COVID-19 Patient Visit Comparisons Using k-Nearest Neighbors Imputation followed by Model Training

| **Comparison** | **Model** | **AUC (95% CI)** | **Sensitivity** | **Specificity** | **Youden Index** |
| --- | --- | --- | --- | --- | --- |
| Year3_vs_Year4 | LightGBM | 0.976 (0.936-1.016) | 0.944 ± 0.046 | 0.557 ± 0.402 | 0.501 ± 0.437 |
|  | XGBoost | 0.979 (0.960-0.998) | 0.925 ± 0.069 | 0.596 ± 0.365 | 0.522 ± 0.410 |
|  | HistGB | 0.979 (0.948-1.010) | 0.962 ± 0.047 | 0.609 ± 0.469 | 0.571 ± 0.504 |
|  | CatBoost | 0.989 (0.974-1.005) | 0.925 ± 0.069 | 0.596 ± 0.365 | 0.522 ± 0.410 |
| Year1_vs_Year4 | LightGBM | 0.994 (0.984-1.004) | 0.927 ± 0.089 | 0.703 ± 0.350 | 0.630 ± 0.424 |
|  | XGBoost | 0.988 (0.976-1.000) | 0.927 ± 0.068 | 0.550 ± 0.385 | 0.477 ± 0.441 |
|  | HistGB | 0.994 (0.984-1.004) | 0.945 ± 0.073 | 0.687 ± 0.372 | 0.632 ± 0.433 |
|  | CatBoost | 0.997 (0.993-1.001) | 0.964 ± 0.045 | 0.662 ± 0.412 | 0.625 ± 0.443 |
| Year2_vs_Year4 | LightGBM | 0.982 (0.956-1.008) | 0.965 ± 0.043 | 0.513 ± 0.534 | 0.478 ± 0.572 |
|  | XGBoost | 0.999 (0.996-1.001) | 0.910 ± 0.053 | 0.625 ± 0.189 | 0.535 ± 0.234 |
|  | HistGB | 0.999 (0.996-1.001) | 0.965 ± 0.043 | 0.623 ± 0.448 | 0.588 ± 0.480 |
|  | CatBoost | 1.000 (1.000-1.000) | 0.983 ± 0.033 | 0.756 ± 0.414 | 0.740 ± 0.430 |
| Year1_vs_Year3 | LightGBM | 0.992 (0.982-1.002) | 0.969 ± 0.038 | 0.626 ± 0.454 | 0.595 ± 0.480 |
|  | XGBoost | 0.992 (0.980-1.004) | 0.938 ± 0.090 | 0.759 ± 0.274 | 0.698 ± 0.337 |
|  | HistGB | 0.995 (0.986-1.004) | 0.969 ± 0.038 | 0.696 ± 0.396 | 0.665 ± 0.417 |
|  | CatBoost | 0.995 (0.987-1.002) | 0.969 ± 0.038 | 0.696 ± 0.396 | 0.665 ± 0.417 |
| Year1_vs_Year2 | LightGBM | 0.987 (0.971-1.002) | 0.946 ± 0.027 | 0.376 ± 0.396 | 0.322 ± 0.416 |
|  | XGBoost | 0.980 (0.955-1.005) | 0.959 ± 0.033 | 0.359 ± 0.500 | 0.318 ± 0.531 |
|  | HistGB | 0.982 (0.957-1.007) | 0.959 ± 0.033 | 0.378 ± 0.486 | 0.337 ± 0.516 |
|  | CatBoost | 0.984 (0.959-1.010) | 0.972 ± 0.034 | 0.525 ± 0.523 | 0.498 ± 0.552 |
| Year2_vs_Year3 | LightGBM | 0.959 (0.909-1.009) | 0.936 ± 0.079 | 0.586 ± 0.491 | 0.522 ± 0.553 |
|  | XGBoost | 0.971 (0.927-1.015) | 0.936 ± 0.079 | 0.605 ± 0.466 | 0.541 ± 0.526 |
|  | HistGB | 0.942 (0.881-1.003) | 0.936 ± 0.079 | 0.605 ± 0.466 | 0.541 ± 0.526 |
|  | CatBoost | 0.967 (0.918-1.016) | 0.936 ± 0.079 | 0.605 ± 0.466 | 0.541 ± 0.526 |

Classification performance metrics following k-nearest neighbors (kNN) imputation of missing values. The table shows AUC with 95% confidence intervals, sensitivity, specificity, and Youden Index across all classifier models and temporal visit comparisons. Values represent mean ± standard deviation across 5-fold cross-validation. Traditional machine learning models (SVM, Naive Bayes, KNN, MLP) demonstrated lower performance (AUC 0.31-0.93) compared to gradient boosting methods (AUC 0.94-1.00), indicating superior capability of ensemble methods for handling imputed multimodal COVID-19 patient data.

# **Supplemental Table 6**. Classification Performance Metrics for Longitudinal COVID-19 Patient Visit Comparisons Using Random Forest Imputation followed by Model Training

| **Comparison** | **Model** | **AUC (95% CI)** | **Sensitivity** | **Specificity** | **Youden Index** |
| --- | --- | --- | --- | --- | --- |
| Year3_vs_Year4 | LightGBM | 0.977 (0.937-1.017) | 0.962 ± 0.047 | 0.609 ± 0.469 | 0.571 ± 0.504 |
|  | XGBoost | 0.980 (0.959-1.002) | 0.925 ± 0.069 | 0.596 ± 0.365 | 0.522 ± 0.410 |
|  | HistGB | 0.991 (0.979-1.003) | 0.962 ± 0.047 | 0.609 ± 0.469 | 0.571 ± 0.504 |
|  | CatBoost | 0.985 (0.961-1.009) | 0.944 ± 0.046 | 0.590 ± 0.368 | 0.534 ± 0.406 |
| Year1_vs_Year2 | LightGBM | 0.987 (0.967-1.008) | 0.946 ± 0.027 | 0.394 ± 0.375 | 0.339 ± 0.395 |
|  | XGBoost | 0.979 (0.954-1.004) | 0.972 ± 0.034 | 0.525 ± 0.523 | 0.498 ± 0.552 |
|  | HistGB | 0.986 (0.970-1.002) | 0.946 ± 0.027 | 0.242 ± 0.375 | 0.188 ± 0.400 |
|  | CatBoost | 0.985 (0.959-1.011) | 0.972 ± 0.034 | 0.525 ± 0.523 | 0.498 ± 0.552 |
| Year1_vs_Year3 | LightGBM | 0.997 (0.994-1.001) | 0.969 ± 0.038 | 0.724 ± 0.339 | 0.693 ± 0.363 |
|  | XGBoost | 0.995 (0.986-1.004) | 0.969 ± 0.038 | 0.724 ± 0.339 | 0.693 ± 0.363 |
|  | HistGB | 0.994 (0.985-1.003) | 0.969 ± 0.038 | 0.696 ± 0.396 | 0.665 ± 0.417 |
|  | CatBoost | 0.998 (0.995-1.002) | 0.969 ± 0.038 | 0.696 ± 0.396 | 0.665 ± 0.417 |
| Year2_vs_Year4 | LightGBM | 0.991 (0.971-1.010) | 0.965 ± 0.043 | 0.741 ± 0.296 | 0.706 ± 0.329 |
|  | XGBoost | 1.000 (1.000-1.000) | 0.948 ± 0.067 | 0.752 ± 0.278 | 0.701 ± 0.331 |
|  | HistGB | 0.991 (0.977-1.006) | 0.965 ± 0.043 | 0.741 ± 0.296 | 0.706 ± 0.329 |
|  | CatBoost | 0.997 (0.992-1.003) | 0.965 ± 0.043 | 0.702 ± 0.367 | 0.668 ± 0.393 |
| Year1_vs_Year4 | LightGBM | 0.999 (0.997-1.001) | 1.000 ± 0.000 | 1.000 ± 0.000 | 1.000 ± 0.000 |
|  | XGBoost | 0.997 (0.991-1.003) | 0.909 ± 0.115 | 0.767 ± 0.261 | 0.676 ± 0.363 |
|  | HistGB | 0.997 (0.991-1.003) | 0.964 ± 0.045 | 0.709 ± 0.392 | 0.673 ± 0.415 |
|  | CatBoost | 0.996 (0.987-1.004) | 0.945 ± 0.109 | 0.810 ± 0.297 | 0.756 ± 0.379 |
| Year2_vs_Year3 | LightGBM | 0.950 (0.871-1.029) | 0.967 ± 0.067 | 0.685 ± 0.489 | 0.652 ± 0.539 |
|  | XGBoost | 0.954 (0.886-1.023) | 0.951 ± 0.066 | 0.605 ± 0.466 | 0.556 ± 0.518 |
|  | HistGB | 0.956 (0.890-1.022) | 0.967 ± 0.067 | 0.685 ± 0.489 | 0.652 ± 0.539 |
|  | CatBoost | 0.972 (0.931-1.013) | 0.967 ± 0.067 | 0.685 ± 0.489 | 0.652 ± 0.539 |

Classification performance metrics following random forest (RF) imputation of missing values. The table displays AUC with 95% confidence intervals, sensitivity, specificity, and Youden Index for all evaluated classifiers across temporal comparisons. Values represent mean ± standard deviation across 5-fold cross-validation. Results demonstrate consistent high performance across imputation strategies, with gradient boosting ensemble methods (LightGBM, XGBoost, HistGB, CatBoost) achieving near-perfect discrimination (AUC > 0.97) for most visit pair comparisons, validating the robustness of the classification pipeline.

# **Supplemental Figure 1 ROC-AUC for classification performance across pairwise visit comparisons using machine learning models.**

# **Supplemental Figure 2** LIME based longitudinal feature importance for classifying the follow-up stage of patient status, computed using gradient boosting classifiers LightGBM and XGBoost after Random Forest-based imputation of missing data.

**(A)**

**(B)**

# **Supplemental Figure 3** SHAP-based longitudinal feature importance for classifying the follow-up stage of patient status, computed using gradient boosting classifiers LightGBM and XGBoost after Random Forest-based imputation of missing data.

**(A)**

**(B)**

# **References**

1. Simren J, Andreasson U, Gobom J, et al. Establishment of reference values for plasma neurofilament light based on healthy individuals aged 5-90 years. Brain Commun. 2022;4(4):fcac174. doi:10.1093/braincomms/fcac174

2. Tybirk L, Hviid CVB, Knudsen CS, Parkner T. Serum GFAP - reference interval and preanalytical properties in Danish adults. Clin Chem Lab Med. Oct 26 2022;60(11):1830-1838. doi:10.1515/cclm-2022-0646
